# Identification of a Large Pool of Microorganisms with an Array of Porphyrin Based Gas Sensors

**DOI:** 10.3390/s16040466

**Published:** 2016-04-01

**Authors:** Nicola M. Zetola, Chawangwa Modongo, Keikantse Matlhagela, Enoch Sepako, Ogopotse Matsiri, Tsaone Tamuhla, Bontle Mbongwe, Eugenio Martinelli, Giorgio Sirugo, Roberto Paolesse, Corrado Di Natale

**Affiliations:** 1 Department of Radiation Oncology, University of Pennsylvania, 3400 Civic Center Blvd, Philadelphia, PA 19104, USA; zetolan@gmail.com; 2 Faculty of Medicine, University of Botswana, Private Bag, Gaborone 0022, Botswana; matlhagelak@mopipi.ub.bw (K.M.); enoch.sepako@mopipi.ub.bw (E.S.); 3 Botswana-University of Pennsylvania Partnership, P.O. Box AC 157 ACH, Gaborone 0022, Botswana; ntungwana@yahoo.co.uk (C.M.); ogopotsem@bup.org.bw (O.M.); tsaone.tamuhla@gmail.com (T.T.); 4 Department of Environmental Science, University of Botswana, Private Bag, Gaborone 0022, Botswana; mbongwe@mopipi.ub.bw; 5 Department of Electronic Engineering, University of Rome Tor Vergata, Via Politecnico 1, Roma 00133, Italy; martinelli@ing.uniroma2.it; 6 Centro di Ricerca, Ospedale San Pietro Fatebenefratelli, Via Cassia 600, Roma 00189, Italy; gsirugo@gmail.com; 7 Department of Chemical Science and Technology, University of Rome Tor Vergata, Via della Ricerca Scientifica, Roma 00133, Italy; roberto.paolesse@uniroma2.it

**Keywords:** microorganisms, volatile compounds, porphyrins, quartz microbalance

## Abstract

The association between volatile compounds (VCs) and microorganisms, as demonstrated by several studies, may offer the ground for a rapid identification of pathogens. To this regard, chemical sensors are a key enabling technology for the exploitation of this opportunity. In this study, we investigated the performance of an array of porphyrin-coated quartz microbalance gas sensors in the identification of a panel of 12 bacteria and fungi. The porphyrins were metal complexes and the free base of a functionalized tetraphenylporphyrin. Our results show that the sensor array distinguishes the VC patterns produced by microorganisms *in vitro*. Besides being individually identified, bacteria are also sorted into Gram-positive and Gram-negative.

## 1. Introduction

Early detection and classification of pathogenic microorganisms is crucial for a rapid treatment initiation and improved clinical outcome. However, classical microbiological assays based on cultures usually require days to be measurable [1] and rapid techniques, such as Gram-stain, have little sensitivity and largely fail in identifying the strain [2]. In order to reduce the measurement time, a number of alternative techniques are being devised [3]. Among them, the analysis of volatile compounds (VCs) is particularly appealing for the relative simplicity of sample collection and analysis [4]. The production of VCs by microorganisms is well known. In food industries, these characteristics are utilized to produce a desired odor during the processes of food transformation.

These compounds emerge as products of metabolic pathways. For instance, the synthesis of fatty acids is thought to be the source of a pattern of alkanes, alcohols, and ketones [5]. Due to the ubiquity and the complexity of metabolic processes, microorganisms produce a wide range of VCs including, besides the above mentioned chemicals, aldehydes, esters and sulfides. Eventually, there is a common pattern of volatile compounds that makes the identification of specific classes of bacteria difficult. On the other hand, some compounds more specific for some class of bacteria have been identified. For instance, fatty acids are found to be abundant in anaerobic bacteria [6], indole is thought to be at the origin of the rotting smell of *Escherichia coli* [7], long-chain alcohols are typical of Gram-negative enteric bacteria [8], and hydrogen cyanide is associated with *Pseudomonas Aeruginosa* [9]. All these studies are strongly dependent on the experimental conditions. In particular, the composition of the culture media interferes with the VC production both in the terms of quantity and quality [10].

The analysis of VCs is also emerging as a diagnostic tool for human pathologies. In the case of complex organisms, VCs are released by a manifold of different mechanisms and pathways. The global composition of VCs is called “volatilome” [11]. The relationship between VCs and pathologies in humans is not only based on the obvious observation that the VCs of microorganisms are simply added to those released by the human body, but also on the fact that pathologies may change the normal metabolic processes. As a consequence, even non infective diseases may be detected measuring the changes in the VC pattern [12]. However, the detection of infections may be facilitated since many of the VCs produced by pathogenic bacteria are not found in the human volatilome. This is the case of common bacteria involved in sepsis and many other serious human diseases such as *Staphylococcus aureus*, *Streptococcus pneumoniae*, *Enterococcus* spp., *Pseudomonas aeruginosa*, *Klebsiella pneumoniae*, and *Escherichia coli* [13].

Most of the studies aimed at establishing the relationship between VCs and microorganisms have been carried out in the realm of analytical chemistry, then using gas separation and identification instrumentation, such as gas-chromatograph mass-spectrometer (GC/MS) [14]. GC/MS may provide the measure of single compounds, making possible, in principle, the identification of bacteria only detecting the alleged species related compounds. Actually, the detection based on few selected compounds may be risky because of the large and unpredictable fluctuations of the concentration between infected and non infected samples. For instance, 2-aminoacetophenone that has been indicated as a biomarker of *Pseudomonas Aeruginosa* is also found in the breath of non infected subjected after the ingestion of cheese or wine [15]. The obvious alternative is to expand the number of considered VCs giving rise to a pattern of volatile compounds. Samples are then described by multivariate data, and the identification of bacteria results from a pattern recognition algorithm [16]. The multivariate patten can emerge assembling data extracted from a GC/MS measurement, selecting a number of relevant compounds or even considering the totality of compounds, namely the total volatilome.

A particular approach to global pattern analysis is provided by the arrays of broadly selective chemical sensors. These are ensembles of gas sensors of limited selectivity but characterized by different sensitivity patterns. The use of sensors is particularly fascinating because sensors are devices that provide a natural bridge between chemistry and electronics enabling the access to technological issues such as miniaturization, low-cost of production, and a natural connection with the realm of computers. It is worth reminding readers that since olfactory receptors are also partially selective [17], the arrays of gas sensors are often called “electronic noses”.

Sensor arrays have been used to discriminate among classes in many different fields [18], and among them medical diagnosis, in particular for identifying bacteria and tumor cells *in vitro* as well as *in vivo* [19]. The sorting of microorganisms has been demonstrated with many different sensor technologies. Arrays of conductive polymers were shown to detect *Mycobacterium tuberculosis* and *Pseudomonas aeruginosa* in sputum [20]. Metal oxide semiconductors were used to identify four non-pathogenic bacteria included two different *Escherichia Coli* [21]. Finally, a combination of metal oxide semiconductors and catalytic metal gated field effect transistors identified ten different bacteria isolated from clinical blood samples [22]. This paper describes the application of an array of porphyrins coated quartz microbalances to the identification of a large pool of bacteria. Porphyrins are primarily known for their role in life processes. Iron complexes, for instance, are prosthetic groups of important proteins such as hemoglobin and cytochromes, while reduced porphyrins, as magnesium chlorine complexes, are fundamental in the photosynthetic processes.

These exceptional functions of natural porphyrins are extended to their synthetic counterparts. Porphyrins indeed are intensively studied for very different applications such as the dye sensitized solar cells or the electronic molecular applications [23].

Porphyrins are among the most suitable molecular platforms for the design of sensor arrays [24]. Their molecular framework offers a wide range of interaction mechanisms for analyte binding, spanning from the weak van der Waals forces to hydrogen bonds, *π* − *π* interactions, and, finally, to the coordination to the central metal ion [25]. Although non selective, the sensitivity of porphyrins can be oriented, through suitable molecular design, towards different classes of analytes. The sensing properties of porphyrins based sensors can be empirically related to the properties of the individual porphyrins. A large part of the interactions can be qualitatively described by the Hard and Soft Acids and Bases (HSAB) Pearson’s rule that states that hard acids prefer hard bases and soft acids preferentially bind with soft basis [26]. For instance, HSAB elucidates the sensitivity of porphyrins coated quartz microbalance sensors to amines and alcohols [27]. Arrays of porphyrins coated quartz microbalances are very efficient in discriminating samples characterized by a wide chemical pattern. This case is met in different fields and, in particular, in medical diagnostics. To this regard, arrays of quartz microbalance sensors have been used to diagnose lung cancer [28] and asthma [29] from breath analysis.

Here, a large pool of 12 microorganisms (both Gram-positive and negative) and one fungus has been investigated. Some of these microorganisms are of particular pathogenic relevance. Results show that the array of porphyrins based sensors is sufficiently sensitive and selective to identify the different species and, in particular, a separation between Gram-negative and Gram-positive bacteria is achieved.

## 2. Experimental Section

This study involved eleven of the most common human pathogenic bacteria: *Staphylococcus aureus*, *Streptococcus saprophyticus*, *Streptococcus typicae*, *Enterococcus*, *Enterococcus faecalis*, *Salmonella enterica*, *Lysteria monocytogenes*, *Morganella morganii*, *Klebsiella pneumoniae*, *Acinetobacter baumanni*, *Salmonella* spp., and one fungus: *Candida* spp.

Microorganisms were grown on agar, and the headspace was sampled from the Petri dishes (92 mm × 16 mm) 48 h after inoculation.

The electronic nose was an ensemble of eight quartz microbalance (QMB) gas sensors. In these sensors, a slight mass change (Δm) on the quartz surface results in frequency changes (Δf) of the electrical output signal of the oscillator circuit, at which each sensor is connected. The quantities Δm and Δf are linearly proportional in the low-perturbation regime [30]. The QMBs had a fundamental frequency of 20 MHz, corresponding to a mass resolution in the order of a few nanograms.

The free base of the 5,10,15,20-tetrakis-(4-butyloxyphenyl)porphyrin (TBPPH_2_) and corresponding metal complexes (TBPPCu, TBPPCo, TBPPZn, TBPPMg, TBPPMnCl, TBPPFeCl, TBPPSnCl_2_) were used to functionalize the QMBs composing the array. TBPPH_2_ and the corresponding metal complexes have been prepared following literature methods [31]. The coordinated metal ions were chosen on the basis of their different HSAB character and their synthetic availability. All porphyrins were functionalized with alkyl chains in order to improve the porphyrins film permeability and then the sensitivity. However, the alkyl chains increase the magnitude of non-specific interactions, reducing the influence of the intrinsic porphyrin selectivity. The butyloxy derivative used here is the optimal compromise between these opposite effects [32].

Thin films of sensing materials were deposited by a spray-coating on both sides of the quartz disks, from 10^−3^ M of porphyrins in CHCl_3_. For each sensor, the total coating resulted in a frequency shift of 60 KHz. The sensors were housed in a stainless steel measurement chamber having a volume of 10 mL. Each sensor was connected to an individual oscillator circuit. Frequencies were measured by means of an integrated frequency counter and then stored on a computer.

Sensors were calibrated measuring their response to a series of compounds representative of different chemical families, which were propionic acid, ethanol, triethylamine, hexane, toluene, and dimethysulfide. Vapors of the volatile compounds were generated by bubbling a N2 stream into a liquid sample of the compounds and diluting it with nitrogen gas. The dilution rate was controlled by a computer-driven four channel mass-flow controller (MKS). The concentration of the volatile compounds was calculated by the Antoine equation using the parameters listed in the database of the National Institute of Technology (NIST) [33]. The VCs of the cultured microorganisms were captured by means of a suitably designed sampler, consisting of a metallic lid fitting the size of the Petri dish. The inlet was switched to the sample for 120 s, and this time was compatible with the amount of headspace in the Petri dish. During each measurement session, the sensors were continually kept under a constant flow of reference air.

**Figure 1:**
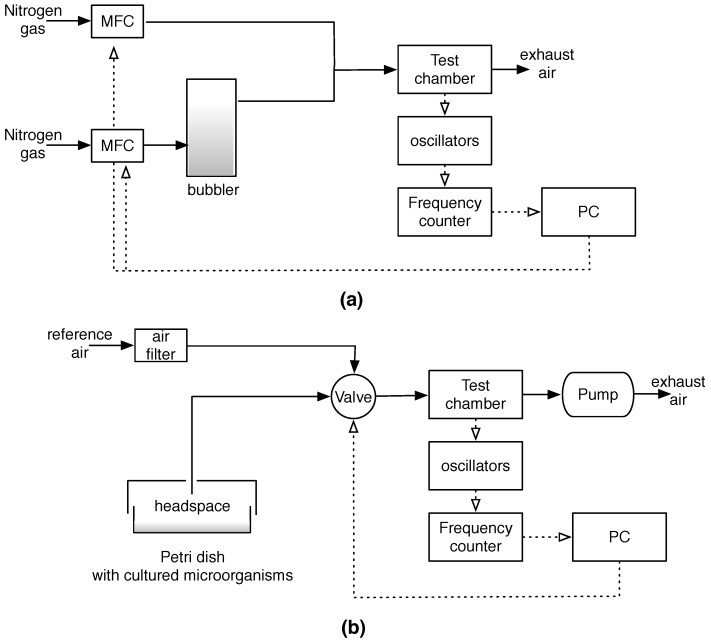
Schematic diagram of the experimental setups used for sensors calibration (**a**) and microorganisms measurements (**b**). All the parts have been hold in a ventilated hood at constant temperature and humidity. Dotted lines indicate the electrical connections.

Figure 1 shows a block-diagram of the experimental setups.

The measurements were performed in a period of approximately 20 days. Some microorganisms were measured in more than one session. Each day, a reference blank culture media was measured. The experiments were performed in the laboratories of the University of Botswana in Gaborone, Botswana.

## 3. Results and Discussion

The array of sensors has been characterized in order to determine the contribution of each sensor to the sensor array. It is known that porphyrins based quartz microbalances are rather non selective, namely these sensors can sense a wide range of different compounds [32]. The sensitivity is expected to be mainly driven by the interactions of the VCs with the metal ion in the porphyrin complexes and with the aromatic system. On the other hand, it is not possible to neglect that other interactions, such as van der Waals forces, strongly depend on the molecular arrangement of the porphyrins in solid phase. The mutual interactions among porphyrins may be altered by the properties of the metal ion. Eventually, the metal ion affects the coordination and the solid state structure of the porphyrin film and both of them contribute to shape the sensor sensitivity.

However, from the array point of view, even the slightest differences between sensors allow for the recognition of different compounds. In order to characterize the array, the sensors have been exposed to vapors of compounds representative of diverse chemical families and then characterized by different functional groups. The selected vapors were propionic acid, dimethylsulfide, triethylamine, hexane, toluene, and ethanol. The saturated vapor of each compound was mixed with a pure nitrogen gas to give rise to different concentrations.

A simple tool to study the contribution of individual sensors to the array is offered by Principal Component Analysis (PCA). PCA is a method to decompose a set of multivariate data into non-correlated variables [34]. In practice, PCA defines a number of novel variables obtained as the linear combination of measurable variables. Since the principal components are non correlated, the total variance of the data set is the sum of the variance of each principal component. The carried variance establishes a hierarchy among the principal components, with the obvious meaning that principal components carrying the largest variance describe a collective behavior of the sensors.

Here, PCA has been calculated on the autoscaled data matrix, namely the data of each sensor have been normalized to zero mean and unitary variance. Before PCA, the data were linearly normalized in order to remove the quantitative information according to the following relationship that is strictly valid under linear relationship between sensor response (Δ*f*) and concentration (c) [35].

(1) $$\Delta f_{i}\to \frac{\Delta f_{i}}{\sum_{k}\Delta f_{k}}=\frac{S_{ij}c_{j}}{\sum_{k}S_{kj}c_{j}}=\frac{S_{ij}}{\sum_{k}S_{kj}}$$

where Δ*f_i_* is the response of the i-th sensor, *S_ij_* is the sensitivity of the sensor i-th to the compound j-th and *c_j_* is the concentration of the j-th compounds. The sum is extended to all the sensors of the array. In this way, the response of each sensor is weighted by the sum of the response for each sensor.

**Figure 2:**
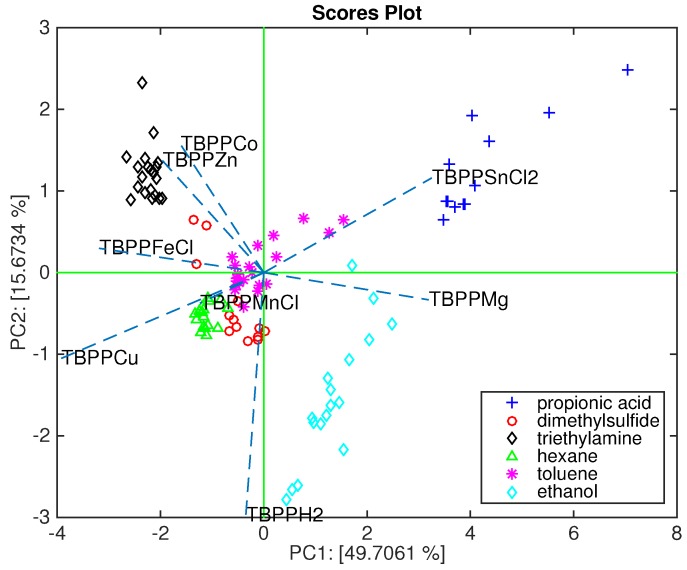
Biplot of the first two principal components where about 65% of the total variance of the calibration data is explained.

Figure 2 shows the biplot of the first and second principal components. In this plot, the array capability to segregate each compound can be appreciated as well as the relation between sensors and compounds. Thus, we can observe that TBPPSnCl_2_, TBPPMg and TBPPH_2_ are oriented towards the acid and the alcohol, TBPPCo and TBPPZn towards the amine, while TBPPFe, TBPPCu, and TBPPMn are more oriented towards the alkane and the sulfide. It is important to keep in mind that the found sensor-volatile compound relationship has to be considered in the frame of this array.

**Figure 3:**
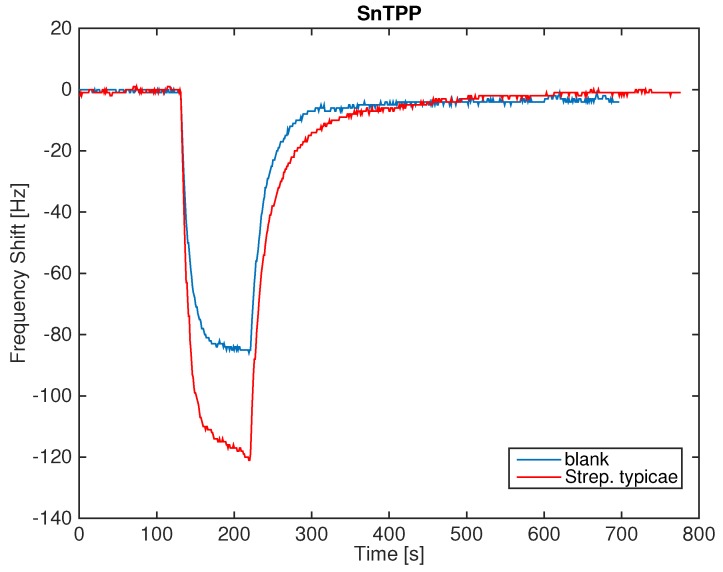
Comparison of the signals (frequency shift) of the sensor coated by TBPPSnCl_2_. In the figure, the responses to the blank culture media and the *Streptococcus saprophyticus* are shown.

Figure 3 shows the signal of one of the sensors in the case of a measurement sequence of the blank culture media headspace and the *Streptococcus saprophyticus* headspace. The exposure to the headspace of the samples produces a more pronounced decrease of the resonant frequency, signaling the absorption of more abundant quantities of molecules. The difference between blank culture media and the bacteria headspace is of the order of 40 Hz. Since the signal noise is of the order of 1 Hz, the presence of bacteria in the culture media gives rise to a sufficiently large signal. The adsorption of VCs is rather reversible and the consequent desorption is approximately three times slower than the adsorption.

The difference between the frequency measured immediately before the exposure and at the end of the exposure is considered the sensor response (Δf). This quantity is used in the rest of the paper to compare the responses of the sensor array to the various samples.

**Figure 4:**
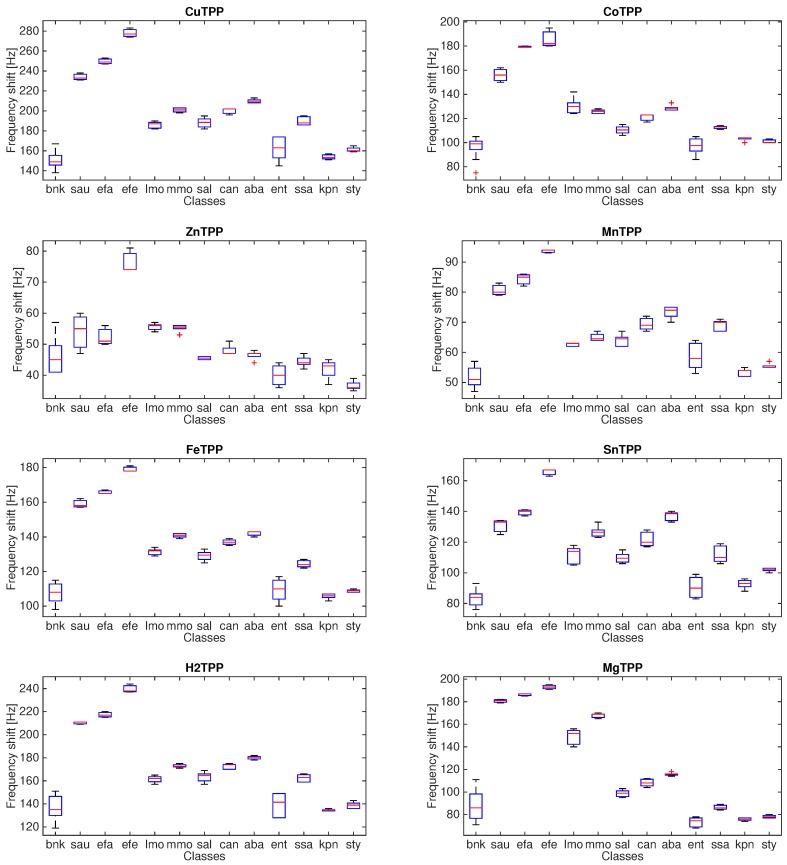
Boxplots of the responses of each sensor to the 13 measured classes. The classes are so labeled: bnk: blank culture; sau: *Staphylococcus aureus*, efa: *Enterococcus faecalis*, efe: *Salmonella enterica*, lmo: *Lysteria monocytogenes*, mmo: *Morganella morganii*, sal: *Salmonella* spp.; can: *Candida* spp., aba: *Acinetobacter baumanni*, ent: Enterococcus, sas: *Streptococcus saprophyticus*, kpn: *Klebsiella pneumoniae*, sty: *Streptococcus typicae*.

Figure 4 shows the statistical distribution of the sensor response towards the different microorganisms and the blank culture.

The dispersion of the measurement of each microorganism is variable and rather homogeneous for all the sensors. There is a common trend among the different sensors. For instance, TBPPCu, TBPPFeCl and TBPPH_2_ share the same relative magnitudes of the responses with respect to the classes. In all sensors, the responses to *Enterococcus* spp., *Klebsiella pneumoniae*, and *Streptococcus typicae* are closer to the blank culture media.

To interpret the sensor signals, it is important to keep in mind that a sensor’s response is the combination of quantitative and qualitative information. Quantitative information relates to the amount of VCs. Qualitative information relates to the kind of compounds. All the analytical studies about the composition of the atmosphere exhaled from microorganisms are rather concerned with the qualitative information, and most of the attention is devoted to the detection of specific compounds for given microorganisms. On the other hand, the strong sensitivity of the sensors to the concentration of VCs makes the sensor responses rather similar to each other. Thus, a plain comparison of the sensor responses, such as that shown in Figure 4, is not sufficient to understand the classification properties of the sensor array. In order to appraise the collective properties of the array, the application of a multivariate data analysis is then necessary. For this scope, the Δf of each sensor of the array is arranged in a vector, and the totality of the measurements forms a matrix, whose rows are the measurements and the columns are the sensors.

In this paper, due to the scarceness of replica per bacteria, the microorganism identification has been studied with an exploratory data analysis performed by Principal Component Analysis (PCA).

**Figure 5:**
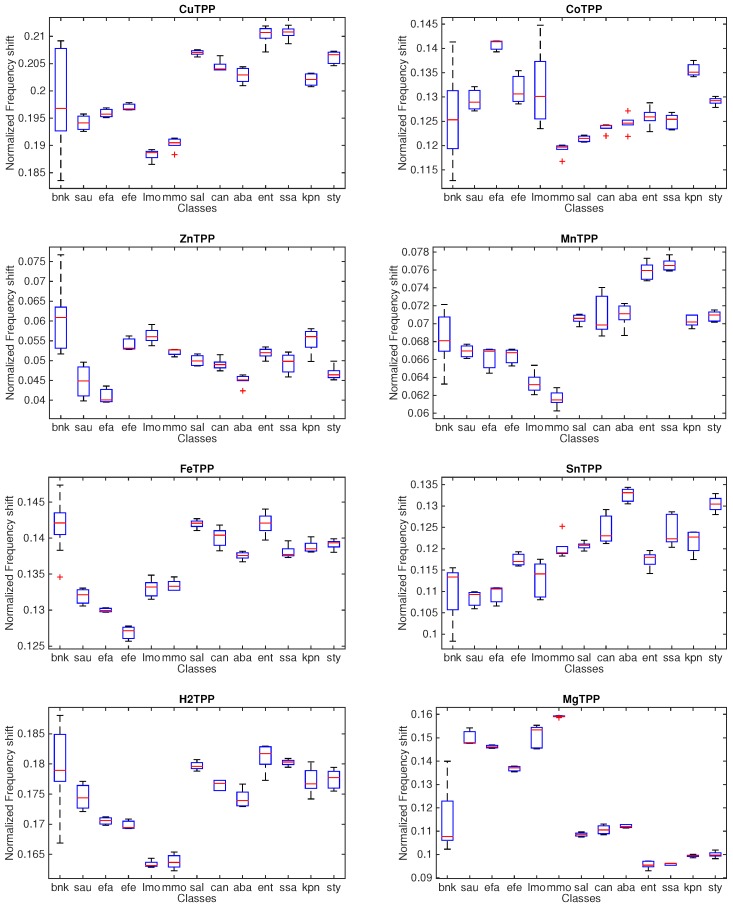
Boxplots of the linearly normalized responses of each sensor to the 13 measured classes. The classes are so labeled: bnk: blank culture; sau: *Staphylococcus aureus*, efa: *Enterococcus faecalis*, efe: *Salmonella enterica*, lmo: *Lysteria monocytogenes*, mmo: *Morganella morganii*, sal: *Salmonella* spp.; can: *Candida* spp., aba: *Acinetobacter baumanni*, ent: *Enterococcus*, sas: *Streptococcus saprophyticus*, kpn: *Klebsiella pneumoniae*, sty: *Streptococcus typicae*.

In this experiment, we have not measured the actual colony-forming unit (CFU) of each culture, so the large correlation of sensors responses shown in Figure 4 may likely be influenced by any non uniformity in bacterial populations. On the other hand, the influence of common modes, such as the concentration of volatile compounds, is expected to be reduced applying Equation (1). Figure 5 shows the statistical distribution of the normalized sensor signals from which it is evident that, after the reduction of common modes, each sensor captures different aspects of the VCs released by microorganisms.

**Figure 6:**
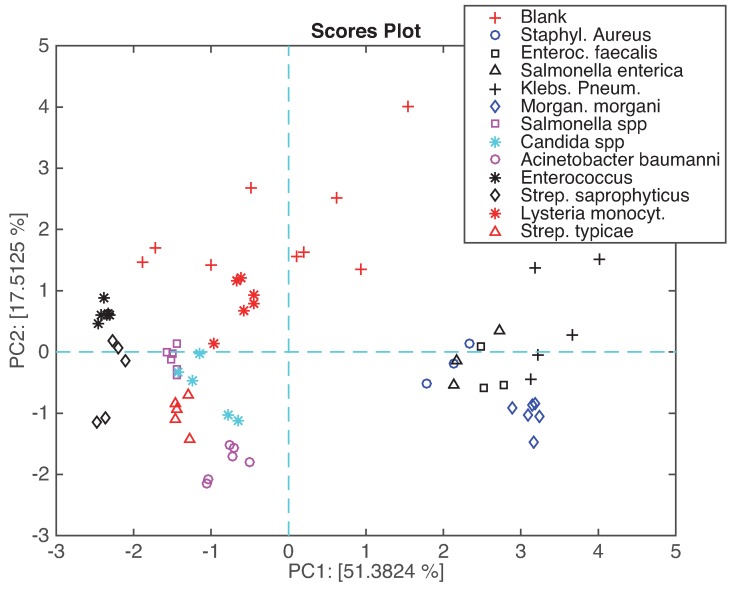
Scores plot of the first two principal components. Samples are labeled according to the microorganisms.

Figure 6 shows the plot of the samples in the plane of the first two principal components. In this plot approximately 68% of the total variance is represented. In the plot, the samples related to the same microorganism are plotted with the same label. The plot shows that the measurements of the same microorganism are rather well reproducible; there is a good separation among the different microorganisms. Few overlaps are shown, for instance *Salmonella enterica* is partially overlapped with *Staphylococcus aureus*. Interestingly, the blank culture media show a larger sparseness with respect to the other classes, suggesting that the presence of the microorganisms elicits a clear signature in the headspace, then less susceptible to fluctuations. To understand this plot, it is important to consider that PC1 (about 51% of total variance) describes the correlated part of the data, and it also contains the residual dependence from the abundance of the headspace. On the other hand, the difference between the blank culture media and the microorganisms is well captured by PC2 (about 17% of variance).

Besides providing a readable plot of the mutual positions of the measured samples, PCA also enables the study of the contribution of the individual sensors to the scores plot. This is obtained plotting the loadings, namely the projection of the original set of the orthogonal axis onto the principal components plane.

**Figure 7:**
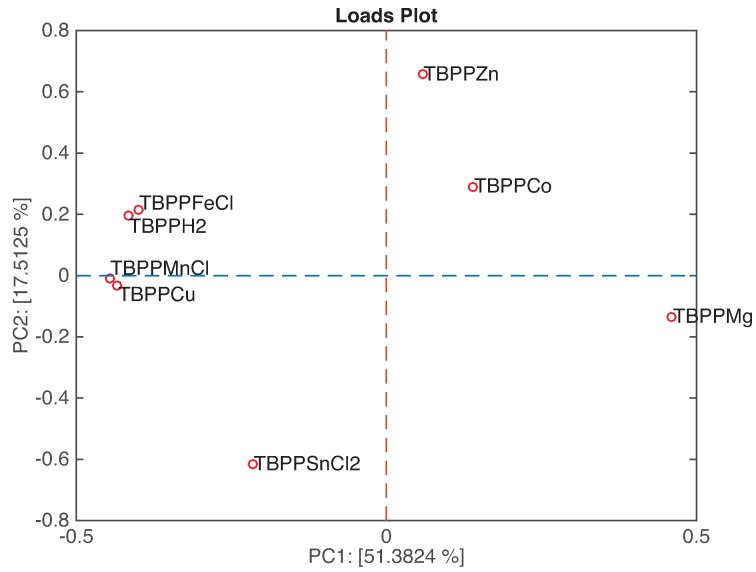
Loadings plot of the first two principal components.

Figure 7 shows the loadings plot related to the first two principal components. In this kind of plot, we can observe the relationship between sensors and, by a comparison with the related scores plot, classes. The loadings of the couples TBPPFeCl and TBPPH_2_, and TBPPMnCl and TBPPCu are overlapped, meaning that these sensors are equally contributing to the plot. The above mentioned couples of sensors and TBPPMg are aligned along PC1, suggesting that these sensors are mostly sensitive to the headspace abundance. On the other hand, TBPPZn, TBPPCo, and TBPPSnCl_2_ are ordered along PC2.

Figure 6 shows that repeated measurements of the same microorganisms are sufficiently reproducible to enable a differentiation among different bacterial categories.

Gram staining is a method of differentiating bacterial species into two large groups: Gram-positive and Gram-negative. Gram staining differentiates bacteria by the chemical and physical properties of their cell walls by detecting peptidoglycan, which is present in a thick layer in Gram-positive bacteria. In a Gram stain test, Gram-positive bacteria retain the dye, while a counterstain added after the crystal violet gives all Gram-negative bacteria a red or pink coloring. In clinical microbiology, the Gram stain is almost always the first step in the identification of a bacterial organism as it correlates with important pathogenic bacterial characteristics [1].

The features of the bacterial membrane may also be instrumental in the composition of the VC patterns and then a relationship between VCs and the Gram staining test may be hypothesized. It was found, for instance, that long-chain alcohols, such as 1-octanol and 1- decanol, are typical of Gram-negative bacteria [14,35].

**Figure 8:**
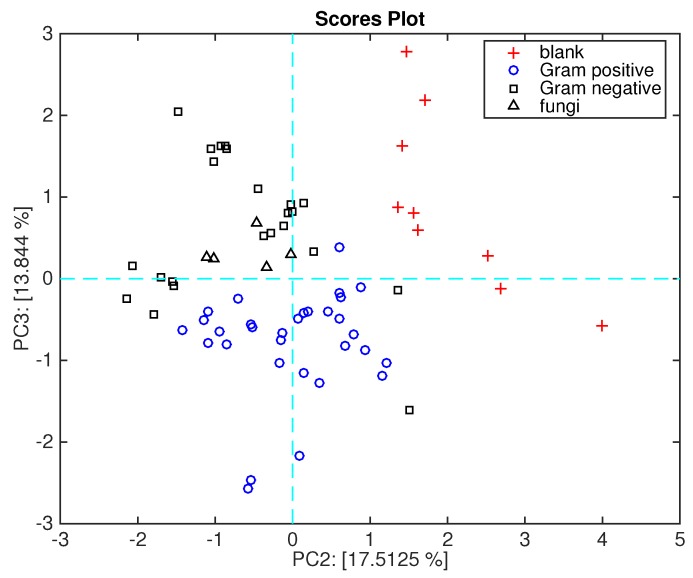
Scores plot of the second and third principal components. Gram-positive and Gram-negative bacteria are labeled.

Figure 8 shows the data plotted in the plane of the second and third principal components. This plot, which explains about 30% of the total variance, shows a net separation between Gram-positive and Gram-negative bacteria. As shown in Figure 3, the second principal component segregates the blank media from the microorganisms, while the second and third plotted together achieve the separation of Gram-positive and Gram-negative bacteria.

**Figure 9:**
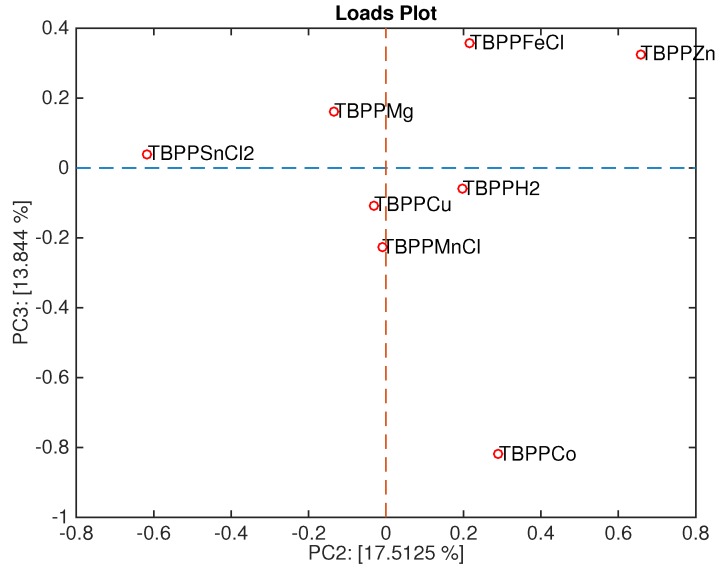
Loadings plot of the second and third principal components.

Figure 9 shows the loadings plot associated with the scores plot of Figure 6. Here, the sensors are ordered in a sequence where TBPPSnCl_2_ and TBPPMg points towards the space region where Gram-negative bacteria are plotted, and TBPPCo towards the opposite region, where Gram-positive bacteria are plotted.

It is interesting to mention that the headspace of Gram-negative bacteria shows an abundance of alcohols [14]. Sensor behavior is in good agreement with the characterization shown in Figure 2 where TBPPMg is oriented towards the semi-plane where alcohol lies, and TBPPSnCl_2_ is oriented towards the acid. It is worth mentioning that acids and alcohols share an OH group and then the sensitivity to acid is somewhat correlated with the sensitivity to alcohols.

A partial least squares discriminant analysis (PLS-DA) classifier was trained and tested to identify the Gram character of the bacteria. In this analysis, the data related to the blank and fungus have been not considered. The training data set contained three Gram-positive bacteria (*Staphylococcus aureus*, *Enterococcus faecalis*, and *Lysteria monocytogenes*) and two Gram-negative bacteria (*Salmonella enterica*, and *Morganella morganii*). The test was performed on the rest of the data set. The classifier was cross-validated with a leave-one-out procedure and the optimal classification was obtained with four latent variables. The classifier achieved 100% of accuracy in both training and test.

## 4. Conclusions

Accurate identification of microorganisms is of critical importance for patient care. Several studies indicate that microorganisms are characterized, *in-vitro*, by a distinctive bouquet of VCs. The development of chemical sensors offers the opportunity to develop simple and affordable devices for a rapid identification of microorganisms. However, among the wide manifold of possible sensing materials and transducers, it is still not defined which sensor is best suited for the scope.

In this study, we investigated the microorganism identification properties of an array of porphyrin coated quartz microbalance sensors. Porphyrins offer a versatile platform for the preparation of chemical sensors. The pyrrolic macrocycle is a sort of a skeleton that can be enriched of functional units, aimed at both improving the sensing properties of the molecule and the preparation of a solid film suitable to be matched with the transducer surface. Here, we considered only one of the many degrees of freedom to differentiate the porphyrin affinities, namely the metal ion complexed in the macrocyclic core. A collection of seven metal complexes and the free base of a substituted tetraphenylporphyrin have been considered.

The array has been applied to the identification of twelve microorganisms (eleven bacteria and one fungus). A multivariate inspection of the array data with the PCA shows that the array can discriminate between the blank culture media and the microorganisms and among the microorganisms. Furthermore, the Gram-negative and Gram-positive bacteria are separated in a proper PCA plot. Related to this, the corresponding loading plot shows that the sensors have more affinity to alcohols and acids, namely molecules carrying an OH group, are more discriminative of Gram-negative species. This is in agreement with the previous literature that reported observation about the abundance of long-chain alcohols in the headspace of Gram-negative bacteria. A PLS-DA classification model trained on five bacteria and tested on the others has shown that it is possible to identify the Gram character, at least in the considered pool of microorganisms, disregarding the strain.

These results are a starting point for the application of a classification algorithm that can actually provide the identification of unknown bacteria cultures, since a classification algorithm, in order to be properly settled, requires an amount of data larger than that collected here and proportional to the number of microorganisms to be identified.
